# Modelling the *Caenorhabditis elegans* gonad over developmental time using the Distal Tip Cell marker *lag-2p::gfp*

**DOI:** 10.17912/micropub.biology.000629

**Published:** 2022-08-17

**Authors:** Matt Crook, Tanya Carvajal, Payton Davis, Jannatul Ferdush, Robert B Page, Elaine Tennyson

**Affiliations:** 1 Texas A&M University-San Antonio

## Abstract

Development is a process that occurs over time, but defects are often scored at the end point of the process being studied. We are interested in understanding the molecular basis of gonad development in *Caenorhabditis elegans* and have used the Distal Tip Cell marker *lag-2p::gfp* to develop a larval size model of gonad growth. We found that gonad length demonstrates two distinct phases relative to larval length, with a breakpoint in mid-L3 stage. We hope that this model will help determine at what point in gonad development our genes of interest act.

**Figure 1. The lag-2p::gfp marker clearly labels the Distal Tip Cells at the leading edge of the developing C. elegans gonad and allows gonad growth to be mapped relative to a proxy for animal length.**
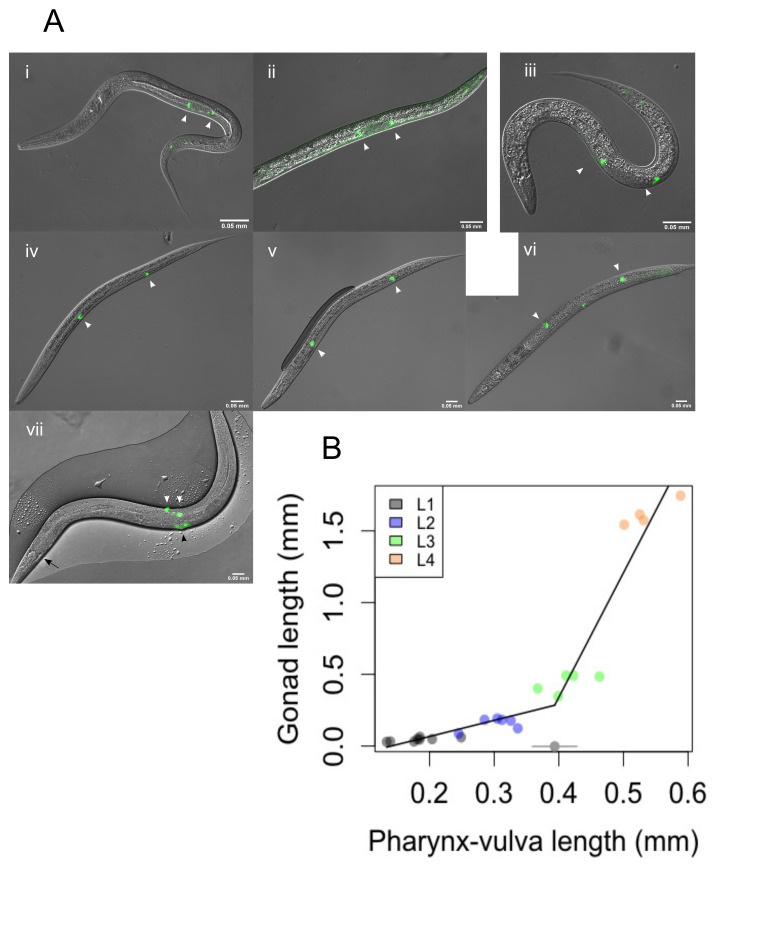
A) A time course series of brightfield DIC/ GFP merged photomicrographs of L1 (i-ii), L2 (iii), L3 (iv+v), L4 (vi) and young adult hermaphrodite (vii). White arrowheads highlight the DTCs in all images. In vii the black arrowhead and arrow point to the vulva/ mid-point and the pharynx-intestine junction, respectively. Animals i through iii were captured at 400x magnification; animals iv through vii were captured at 200x magnification. Scale bar is 50 microns. Head is to the left and ventral to the bottom in all images. B) Total gonad length from DTC to DTC in mm by pharynx-vulval length (PVL) in mm. Circles are individual animals, coloured by stage. The piecewise regression model has been overlaid as a thick black line using the *segmented* R package (Muggeo 2008). The breakpoint and associated 95% confidence interval are shown in grey at the bottom of the graph.

## Description

Gonad development in the free-living nematode *Caenorhabditis elegans* is highly stereotyped and this, along with the transparent nature of *C. elegans* , makes it an excellent model to study organogenesis. The gonad begins development in the first larval stage (L1) at the mid-point of the animal as the gonadal primordium (GP), consisting of four cells, Z1-4. Z1 and Z4 will become the somatic gonad and Z2 and Z3 the germline (Kimble and Hirsh 1979). Through the L1 to L3 stages the two developing gonad arms grow away from the mid-point towards the anterior and posterior, led by the Distal Tip Cells (DTCs) (Hedgecock *et al.* 1987). In late-L3 stage, the gonad tips stop moving away from the mid-point and move dorsally, before extending back towards the mid-point during the L4 stage. By late L4 the DTCs of the two gonad arms end up alongside each other, dorsal to the vulva. After the final moult into adulthood each gonad arm differentiates into the distal gonad (relative to the vulva), the proximal gonad where oocytes form, the spermatheca containing sperm produced during the L4 stage and the uterus where fertilized eggs are stored until they pass through the vulva (Kimble and Hirsh 1979; Hedgecock *et al.* 1987).

We wish to study the molecular control of gonad development by knocking down genes with RNAi. However, by looking at only the endpoint in gonad development in late L4 larvae and young adult hermaphrodites we cannot tell when our phenotype of interest is expressed. Therefore we mapped the development of the *C. elegans* gonad over developmental time using an integrated *lag-2p::gfp* fluorescence marker (JK2868 (qIs56 ( *lag-2p::gfp* + *unc-119* (+)) V), (Blelloch *et al.* 1999)). The qIs56 marker has been used extensively to study early gonad development (Siegfried and Kimble 2002), DTC specification (Asahina *et al.* 2006, Kostic *et al.* 2003, Large and Mathies 2010) and gonad migration defects (Cha *et al.* 2012, Chesney *et al.* 2009, Huang *et al.* 2014) as *lag-2* is a Delta-Serrate-Lag ligand that is strongly expressed only in the DTCs in the gonad (Henderson *et al.* 1994; Chesney *et al.* 2009). However, it does not appear that the *lag-2p::gfp* marker has been used to map gonad development over time.

*C. elegans* strain JK2868 animals were grown on NGM plates seeded with *Escherichia coli* OP50 at 20C. Animals of the approximate stage we wished to image were picked from these plates onto agarose pads and immobilised with 2% tricaine (Sigma-Aldrich, Missouri), which induces flaccid paralysis and did not result any observable shortening of the animals (Wayson *et al.* 1976). We used a Nikon Eclipse Ni epifluorescence microscope and CoolSnap DYNO camera to capture brightfield and GFP images of animals from L1 to young adult, then merged each pair of images using the NIS Elements software (Nikon, New York). An image of a 1mm micrometer scale was also captured at 200x and 400x magnification to determine the Set Scale value in ImageJ. For each animal the developing gonad was measured in ImageJ from the tip of one DTC along the length of the gonad to the tip of the second DTC. If only one gonad arm was fully visible it was measured from DTC to vulva and the measurement doubled. Any images of animals with gonad migration defects, which occurs at up to 25% in JK2868 (Cha *et al.* 2012), were discarded and not used. Given that the qIs56 transgene was almost certainly outcrossed several times in the process of making strain JK2868 one must assume that these mild gonad migration defects (Cha *et al.* 2012) are the result of transgene expression, not background mutations from the integration process. As such we would strongly advise determining the type and penetrance of any gonad development phenotype relative to wild type first, before using this transgene to examine the timing of that phenotype. Empty vector or *unc-22* RNAi controls will also help establish the background level of gonad migration defects in the qIs56-containing background. To generate a quantitative measure of larval growth, the distance from the pharynx-intestine junction to either the midpoint of the gonad (L1 and L2) or the center of the vulva (L3 and L4) was measured, which is referred to as the Pharynx-Vulva Length (PVL). We then used the R package *segmented* (Muggeo 2008) to fit a piecewise linear model that treated gonad length as the response and PVL as a predictor. We used the Bayesian information criterion (BIC) to determine the number of breakpoint parameters to estimate *via* the ‘selgmented’ function. The 95% confidence intervals for the two slopes and the break point were determined using the ‘slope’ and ‘lines.segmented’ functions, respectively. All statistical analyses were carried out using R, version 4.1.2 ( www.r-project.org ).

*lag-2p::gfp* proved to be an excellent marker, labelling the DTCs in all larval stages, with expression also seen in L4 and adult vulvas (Fig. 1a) as described previously (Large and Mathies 2010, Zhang and Greenwald 2011). When gonad length as a function of pharynx-vulval length (PVL) was analysed BIC indicated that a model with a single break point was better than models with 0, 2, or 3 breakpoints. (Fig. 1b). The point estimate for the breakpoint was a PVL of 0.394 mm and the 95% confidence interval for this estimate is shown in Fig. 1c. The estimate for the intercept was -0.1578 and the standard error of this parameter estimate was 0.1307. The slope of the first line segment was 1.1234 and the 95% confidence interval for this parameter estimate was 0.04134-2.2054. The estimate for the slope of the second line segment was 8.6760 and the 95% confidence interval for this parameter estimate was 6.9492-10.4030. However, readers are encouraged to interpret these confidence intervals with caution due to the non-normal error variance associated with the fitted model. The breakpoint and increase in slope of the second line segment corresponds to the turn of the gonad arms in late-L3, after which gonad length increases at a faster rate relative to pharynx-vulval length. Our model will allow us to compare gonad development over time between wild type and RNAi knockdowns to discover at which developmental time point errors occur.

## Reagents

JK2868 (qIs56 ( *lag2-p::gfp* + *unc-119* (+)) V)

## References

[R1] Blelloch R, Anna-Arriola SS, Gao D, Li Y, Hodgkin J, Kimble J (1999). The gon-1 gene is required for gonadal morphogenesis in Caenorhabditis elegans.. Dev Biol.

[R2] Chesney MA, Lam N, Morgan DE, Phillips BT, Kimble J (2009). C. elegans HLH-2/E/Daughterless controls key regulatory cells during gonadogenesis.. Dev Biol.

[R3] Hedgecock EM, Culotti JG, Hall DH, Stern BD (1987). Genetics of cell and axon migrations in Caenorhabditis elegans.. Development.

[R4] Henderson ST, Gao D, Lambie EJ, Kimble J (1994). lag-2 may encode a signaling ligand for the GLP-1 and LIN-12 receptors of C. elegans.. Development.

[R5] Holm, S., 1979 A Simple Sequentially Rejective Multiple Test Procedure. Scandinavian Journal of Statistics 6 **:** 65-70.

[R6] Kimble J, Hirsh D (1979). The postembryonic cell lineages of the hermaphrodite and male gonads in Caenorhabditis elegans.. Dev Biol.

[R7] Muggeo, M. R., 2008 Segmented: an R package to fit regression models with broken-line relationships. R News 8 **:** 20-25.

[R8] Siegfried KR, Kimble J (2002). POP-1 controls axis formation during early gonadogenesis in C. elegans.. Development.

[R9] Asahina M, Valenta T, Silhankova M, Korinek V, Jindra M (2006). Crosstalk between a nuclear receptor and beta-catenin signaling decides cell fates in the C. elegans somatic gonad.. Dev Cell.

[R10] Kostić I, Li S, Roy R (2003). cki-1 links cell division and cell fate acquisition in the C. elegans somatic gonad.. Dev Biol.

[R11] Large EE, Mathies LD (2009). hunchback and Ikaros-like zinc finger genes control reproductive system development in Caenorhabditis elegans.. Dev Biol.

[R12] Huang TF, Cho CY, Cheng YT, Huang JW, Wu YZ, Yeh AY, Nishiwaki K, Chang SC, Wu YC (2014). BLMP-1/Blimp-1 regulates the spatiotemporal cell migration pattern in C. elegans.. PLoS Genet.

[R13] Cha DS, Hollis SE, Datla US, Lee S, Ryu J, Jung HR, Kim E, Kim K, Lee M, Li C, Lee MH (2012). Differential subcellular localization of DNA topoisomerase-1 isoforms and their roles during Caenorhabditis elegans development.. Gene Expr Patterns.

[R14] Zhang X, Greenwald I (2011). Spatial regulation of lag-2 transcription during vulval precursor cell fate patterning in Caenorhabditis elegans.. Genetics.

[R15] Wayson KA, Downes H, Lynn RK, Gerber N (1976). Studies on the comparative pharmacology and selective toxicity of tricaine methanesulfonate: metabolism as a basis of the selective toxicity in poikilotherms.. J Pharmacol Exp Ther.

